# Central Nervous System Infection Diagnosis by Next-Generation Sequencing: A Glimpse Into the Future?

**DOI:** 10.1093/ofid/ofx046

**Published:** 2017-03-04

**Authors:** Nguyen Thi Hoang Mai, Nguyen Hoan Phu, Le Nguyen Truc Nhu, Nguyen Thi Thu Hong, Nguyen Ho Hong Hanh, Lam Anh Nguyet, Tran My Phuong, Angela McBride, Do Quang Ha, Ho Dang Trung Nghia, Nguyen Van Vinh Chau, Guy Thwaites, Le Van Tan

**Affiliations:** 1 Oxford University Clinical Research Unit, Ho Chi Minh City, Vietnam; 2 Hospital for Tropical Diseases, Ho Chi Minh City, Vietnam; 3 Vietnam National University, Ho Chi Minh City, Vietnam; 4 Pham Ngoc Thach University, Ho Chi Minh City, Vietnam; 5 Centre for Tropical Medicine, Nuffield Department of Medicine, University of Oxford, United Kingdom

**Keywords:** deep sequencing, Japanese encephalitis virus, Vietnam

## Abstract

Japanese encephalitis virus was detected by deep sequencing for the first time in urine of a 16-year-old boy with encephalitis. Seroconversion and polymerase chain reaction analysis confirmed the metagenomics finding. Urine is useful for diagnosis of flaviviral encephalitis, whereas deep sequencing can be a panpathogen assay for the diagnosis of life-threatening infectious diseases.

On September 16, 2016, a healthy 16-year-old boy from the Mekong Delta in Southern Vietnam suddenly became ill with high fever and rigors, followed by thoracic back pain, diarrhea, and limb weakness. He was admitted to our hospital on September 18, 2016 fully conscious but with high fever (39°C), neck stiffness, and flaccid paralysis of all 4 limbs with absent reflexes. Analysis of the admission cerebrospinal fluid (CSF) showed 93 × 10^3^/mL leucocytes with lymphocyte predominance (79%), elevated protein (0.62 g/L), and normal glucose and lactate concentrations.

One day after admission, he developed fluctuating heart rate and blood pressure and an intermittent erythematous skin rash, indicating severe autonomic dysfunction. His conscious level dropped, and on the fourth day of admission he had focal convulsions of the left arm and left face. He was intubated and mechanically ventilated, and he received phenobarbital, bisoprolol, and high-dose methylprednisone.

## METHODS AND RESULTS

**Figure 1.**
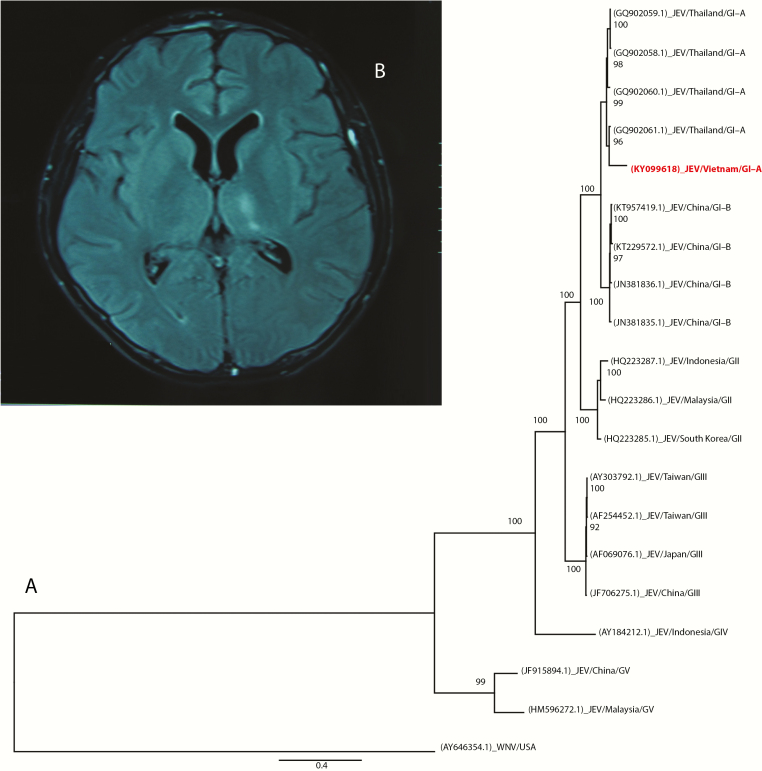
(A) Maximum likelihood tree based on the 1.7-kb sequence obtained from the patient’s urine sample (KY099618) and the corresponding ribonucleic acid (RNA)-dependent RNA polymerase coding region genomic region of representatives of Japanese encephalitis virus (JEV): West Nile virus (WNV) was used as an outgroup. Sequence accession numbers are in brackets. (B) Brain magnetic resonance imaging showing a lesion of the putamen with a high signal on a fluid-attenuated inversion recovery image. Abbreviations: GI-A, genotype I-A; GI-B, genotype I-B; GII, genotype II; GIII, genotype III; GIV, genotype IV; GV, genotype V.

All initial microbiological investigations were negative, including a human immunodeficiency virus test, CSF microscopy for bacteria, fungi, and acid-fast bacteria, bacterial culture (including Mycobacterium tuberculosis), serological testing for Japanese encephalitis virus (JEV)-specific immunoglobulin (Ig)M, and herpes simplex virus polymerase chain reaction (PCR) analysis of the admission CSF. Due to ongoing diagnostic uncertainty, we ordered additional investigations of the admission CSF, rectal swabs collected on the second day of admission, and urine and plasma both taken on the third day of admission. The additional analyses included (1) generic enterovirus reverse transcription (RT)-PCR of CSF and rectal swab, (2) RT-PCR of the CSF, urine, and plasma for Zika and dengue viruses, and (3) dengue virus NS1 rapid test of the CSF, urine, and plasma, but all were negative. Therefore, we decided to perform deep sequencing analysis of viral nucleic acids extracted from the CSF, plasma, urine, and rectal swab specimens using an in-house nonribosomal random PCR and Illumina MiSeq-based assay [1] (Supplementary Data). Subsequently, analysis of the obtained nucleic acid sequences using a publicly available metagenomic pipeline [2] revealed the presence of JEV ribonucleic acid (RNA) in the urine sample. No additional viral pathogens were detected in the other specimens. Phylogenetic analysis of the obtained JEV RNA sequence revealed a close relationship with other regional JEV strains (Figure 1A). The presence of JEV in the original urine was subsequently confirmed by 2 independent JEV RT-PCR assays [3, 4], whereas an attempt at virus isolation was unsuccessful. Japanese encephalitis virus RNA was absent in the admission CSF and the plasma taken on the same day with the urine [3, 4].

Given these results, the second CSF, serum, and urine samples were collected on day 15 of hospitalization. Both CSF and serum samples were JEV IgM positive; urine JEV RT-PCR was now negative. Retrospectively tested, JEV IgM was absent in the plasma sample collected on the third day of admission. Brain magnetic resonance imaging taken on day 28 of hospitalization revealed bilateral lesions of the putamen with high signals on T2-weighted and fluid-attenuated inversion recovery images (Figure 1B), whereas cervical spine magnetic resonance imaging was normal. The patient regained consciousness on day 9 of hospitalization, and limb strength was slowly returning. The patient remained in our hospital until November 9, 2016, when he was transferred to a rehabilitation hospital. At transfer he was conscious and orally fed, but he still had marked weakness of all limbs.

## DISCUSSION

Despite the availability of a vaccine, JEV remains the most common cause of encephalitis, especially in children, across the Asia-Pacific region [5]. Japanese encephalitis virus encephalitis often presents with fever, headache, a reduced level of consciousness and convulsions. An acute flaccid paralysis is a well described feature of JEV infection [5]. Diagnosis is dependent upon serological testing of CSF, demonstrating the presence of JEV-specific IgM, although samples taken early in the illness may be negative, and the assay is usually unavailable outside major centers. The virus can only be detected in CSF by RT-PCR very early in the illness and is rarely helpful in diagnosis [5].

The detection of virus in urine has been reported for other members of the *Flaviviridae* family, such as West Nile, dengue, yellow fever, and, most recently, Zika viruses [6]. Japanese encephalitis virus RNA has previously been detected in urine of infected mice [7], but not humans. A study from China reported negative urine JEV RT-PCR in 52 children serologically confirmed with JEV [8]. Therefore, to the best of our knowledge, this is the first report of JEV detected in urine by deep sequencing and subsequently confirmed by RT-PCR and serology.

As exemplified by the recent Zika virus outbreak, the causes of central nervous system infections can change quickly over time. Rapidly changing epidemiology can challenge diagnostic laboratories offering pathogen-specific assays, especially in resource-limited settings, leaving the cause of many brain infections unknown. In this context, next-generation sequencing technologies have emerged as promising single, panpathogen assays for clinical microbiology and public health, although their clinical utility has yet to be fully defined [9]. These methods may be particularly useful when the pathogen in the tested sample is unexpected or unknown. In addition, the obtained sequences may be used to (1) identify genes conferring antimicrobial resistance, (2) study pathogen evolution, and (3) investigate the sources of outbreaks/epidemics [9].

## CONCLUSIONS

The present case highlights 2 important lessons. First, urine can be a valuable diagnostic specimen when determining the viral cause of encephalitis in flavivirus-endemic regions. Second, next-generation sequencing and viral metagenomics can improve the clinical care of patients with brain infections by enabling an unbiased investigation of viral cause. However, the current high cost of next-generation sequencing and the need for advanced bioinformatic expertise are major barriers to widespread clinical implementation, especially in resource-limited settings. Yet, it is in these regions, where the burden of infectious diseases and the risk of emerging pathogens are highest, that the benefit of these new technologies will be felt most. Well designed prospective studies are urgently needed to assess the ability of next-generation sequencing technologies to transform the clinical microbiology laboratory and the diagnosis of life-threatening infectious diseases.

## Supplementary Data

Supplementary materials are available at *Open Forum Infectious Diseases* online. Consisting of data provided by the authors to benefit the reader, the posted materials are not copyedited and are the sole responsibility of the authors, so questions or comments should be addressed to the corresponding author.

## Supplementary Material

ofx046_suppl_Supplementary_Data
