# Supplementary material for: Central Nervous System Infection Diagnosis by Next-Generation Sequencing: A Glimpse Into the Future?
Source: Open Forum Infect Dis. 2017 Mar 4;4(2):ofx046. doi: 10.1093/ofid/ofx046 (PMC5411956; doi:10.1093/ofid/ofx046)
Supplement: ofx046_suppl_Supplementary_Data [file ofx046_suppl_supplementary_data.docx]

**SUPPLEMENTRAY DATA**

**Central nervous system infection diagnosis by next generation sequencing: a glimpse into the future?**

Nguyen Thi Hoang Mai^1^, Nguyen Hoan Phu^2^, Le Nguyen Truc Nhu^1^, Nguyen Thi Thu Hong^1^, Nguyen Ho Hong Hanh^3^, Lam Anh Nguyet^1^, Tran My Phuong^2^, Angela McBride^1^, Do Quang Ha^1^, Ho Dang Trung Nghia^4^, Nguyen Van Vinh Chau^2^, Guy Thwaites^1,5^ and Le Van Tan^1^

^1^Oxford University Clinical Research Unit, Ho Chi Minh City, Vietnam

^2^Hospital for Tropical Diseases, Ho Chi Minh City, Vietnam

^3^Vietnam National University, Ho Chi Minh City, Vietnam

^4^Pham Ngoc Thach University, Ho Chi Minh City, Vietnam

^5^Centre for Tropical Medicine, Nuffield Department of Medicine, University of Oxford, Oxford, UK

**Methods**

**Viral metagenomics**

Viral metagenomics was carried out on acute CSF, plasma, urine and rectal swab using a previously developed in-house non-ribosomal random PCR and MiSeq based assay [[1](#_ENREF_1)] with some slight modifications. Details are as below.

*Sample pretreatment and nucleic acid extraction*

Prior to nucleic acid isolation 100μl of clinical samples (including CSF, plasma, urine and rectal swab) was treated with 20U of turbo DNase (Ambion, Life Technology, Carlsbad, CA, USA) and 50U of RNase I (Ambion) at 37^o^C for 30 min. Viral RNA was then extracted from the nuclease treated materials using QIAamp viral RNA kit (QIAgen GmbH, Hilden, Germany), following manufacturer’s instructions, and finally eluted in 50μl of elution buffer (provided with the extraction kit).

*cDNA and double stranded DNA synthesis*

Double stranded (ds) DNA was synthesized from the extracted RNA using FR26RV-Endoh primer. Primer sequences can be found elsewhere [[1](#_ENREF_1)]. Firstly, 10μl of extracted RNA was mixed with 0.1μM of the primer and 0.5nM of dNTPs (Roche Diagnostics GmbH, Mannheim, Germany). The mixture was incubated at 65^o^C for 5 min, and was then immediately chilled on ice for 1 min. Secondly, 7μl of a reaction mix containing 200U of Super Script III, 40 U of RNase OUT, 0.1M DTT and 1X first strand buffer (Invitrogen, Carlsbad, CA, USA) was added into the first reaction mixture. The reaction was continued at 25^o^C for 10 min, 37^o^C for 1 min and 94^o^C for 2 min, and then immediately chilled on ice for 2 min. Next, 5U of exo-Klenow fragment (Ambion) and 10U of Ribonuclease H (Ambion) were added into the reaction mixture, which was finally subjected to a ds DNA synthesis step consisting of 25^o^C for 5 min, 37^o^C for 1h and 75^o^C for 10 min.

*Random amplification*

The resulting dsDNA were amplified using FR20RV primer (5’-GCCGGAGCTCTGCAGATATC-3’). PCR amplification was carried out in a total reaction volume of 50μl consisting of 3μl of dsDNA, 0.4μM of primer FR20RV and 45μl of Platinum PCR supermix high fidelity (Invitrogen). The thermal cycling condition consisted of 94^o^C for 2 min and followed by 40 cycles of 94^o^C for 30s, 55^o^C for 30s and 72^o^C for 3min and 1 cycle of 72^o^C for 2min.

*Next generation sequencing library preparation and sequencing*

The resulting rPCR products were purified with use of QIAquick PCR purification kit (QIAgen GmbH, Hilden, Germany) following manufacturer’s instructions. DNA concentration of the purified products was measured by Qubit dsDNA HS kit (Invitrogen). One nanogram of the purified DNA was then subjected to library preparation steps by using Nextera XT DNA library preparation kit (Illumina, San Diego, CA, USA), according to manufacturer’s instructions. Prior to sequencing, the quantity of the prepared library was measured by using KAPA Library Quant Kit (Kapa Biosystems, Wilmington, MA, USA), following manufacturer’s instructions.

The prepared library was sequenced using MiSeq reagent kit V3 in an Illumina Miseq platform (Illumina). For each run, 96 samples were multiplexed and differentiated by double indexes using Nextera XT Index Kit (Illumina).

**Sequence analysis**

The sequences generated by Illumina Miseq were processed to remove primer sequences using Geneious 8.1.5 (Biomatters, San Francisco, CA, USA), and were then subjected to a publicly available metagenomics pipeline namely Taxonomer [[2](#_ENREF_2)] to search for the presence of viral sequences in the corresponding library.

Sequence assembly was carried out by using a reference-based mapping strategy available in Geneious (JEV reference genome sequence: GQ902061.1), followed by manual editing of the obtained consensus.

Pairwise alignment was performed using MUSCLE [[3](#_ENREF_3)] available in Geneious. Phylogenetic reconstruction was performed using maximum likelihood method (ML) with general time reversible (GTR) nucleotide substitution model available in Geneious package, and support for individual nodes was assessed using a bootstrap procedure (1000 replicates).

Metagenomics data of the urine sample was deposited to NCBI (GenBank) under the accession number xxx.

**References**

1. Nguyen, A.T., T.T. Tran, V.M. Hoang, et al., *Development and evaluation of a non-ribosomal random PCR and next-generation sequencing based assay for detection and sequencing of hand, foot and mouth disease pathogens.* Virol J, 2016. **13**: p. 125.

2. Flygare, S., K. Simmon, C. Miller, et al., *Taxonomer: an interactive metagenomics analysis portal for universal pathogen detection and host mRNA expression profiling.* Genome Biol, 2016. **17**(1): p. 111.

3. Edgar, R.C., *MUSCLE: multiple sequence alignment with high accuracy and high throughput.* Nucleic Acids Res, 2004. **32**(5): p. 1792-7.
